# Integrating multimodal ultrasound imaging and machine learning for predicting luminal and non-luminal breast cancer subtypes

**DOI:** 10.3389/fonc.2025.1558880

**Published:** 2025-10-08

**Authors:** Yan Fu, Huang Jing Chen, Hao Zhang, Dong Jie Liu, Xi Chen, Cheng Yu Qiu, Wen Wu Lu, Hao Miao Bai, Qiu Wei Li, Guo Xue Li, Zi Jun Shen, Chang Jiang Gu, Yuan Peng Zhang, Xue Jun Ni

**Affiliations:** ^1^ Department of Ultrasound, Affiliated Hospital of Nantong University, Nantong, Jiangsu, China; ^2^ Department of Medical Informatics, School of Medicine, Nantong University, Nantong, Jiangsu, China; ^3^ Department of Ultrasound, The First Affiliated Hospital of Anhui Medical University, Hefei, Anhui, China; ^4^ Department of General Surgery, Affiliated Hospital of Nantong University, Nantong, Jiangsu, China

**Keywords:** breast cancer, machine learning, ultrasound diagnostics, radiomics, molecular subtypes, multimodal imaging, automated breast volume scanner (ABVS)

## Abstract

**Rationale and Objectives:**

Breast cancer molecular subtypes significantly influence treatment outcomes and prognoses, necessitating precise differentiation to tailor individualized therapies. This study leverages multimodal ultrasound imaging combined with machine learning to preoperatively classify luminal and non-luminal subtypes, aiming to enhance diagnostic accuracy and clinical decision-making.

**Methods:**

This retrospective study included 247 patients with breast cancer, with 192 meeting the inclusion criteria. Patients were randomly divided into a training set (134 cases) and a validation set (58 cases) in a 7:3 ratio. Image segmentation was conducted using 3D Slicer software, adhering to IBSI-standardized radiomics feature extraction. We constructed four model configurations—monomodal, dual-modal, trimodal, and four-modal—through optimized feature selection. These included monomodal datasets comprising 2D ultrasound (US) images, dual-modal datasets integrating 2D US with color Doppler flow imaging (CDFI) (US+CDFI), trimodal datasets incorporating strain elastography (SE) alongside 2D US and CDFI (US+CDFI+SE), and four-modal datasets combining all modalities, including ABVS coronal imaging (US+CDFI+SE+ABVS). Machine learning classifiers such as logistic regression (LR), support vector machines (SVM), AdaBoost (adaptive boosting), random forests(RF), linear discriminant analysis(LDA), and ridge regression were utilized.

**Results:**

The four-modal model achieved the highest performance (AUC: 0.947, 95% CI: 0.884-0.986), significantly outperforming the monomodal model (AUC 0.758, ΔAUC +0.189). Multimodal integration progressively enhanced performance: trimodal models surpassed dual-modal and monomodal approaches (AUC 0.865 vs 0.741 and 0.758), and the four-modal framework showed marked improvements in sensitivity (88.4% vs 71.1% for monomodal), specificity (92.7% vs 70.1%), and F1 scores (0.905).

**Conclusion:**

This study establishes a multimodal machine learning model integrating advanced ultrasound imaging techniques to preoperatively distinguish luminal from non-luminal breast cancers. The model demonstrates significant potential to improve diagnostic accuracy and generalization, representing a notable advancement in non-invasive breast cancer diagnostics.

## 1 Introduction

Breast cancer remains the leading cause of cancer-related mortality among women worldwide (1). It encompasses several molecular subtypes with distinct differences in treatment responses and prognoses, making accurate subtype identification essential for personalized therapy. The luminal subtype, characterized by high levels of estrogen (ER) and progesterone (PR) receptors, generally responds well to hormonal therapies (2). Conversely, non-luminal subtypes, including HER2 (human epidermal growth factor receptor 2)-enriched and triple-negative breast cancers(TNBC), exhibit aggressive behavior, reduced hormonal treatment responsiveness, and poorer outcomes (3, 4). Luminal cancers typically undergo endocrine therapy ± surgery, whereas Non-luminal subtypes require neoadjuvant chemotherapy—a decision that must be made preoperatively. In resource-limited settings, patients wait >2 weeks for IHC results, delaying time-critical therapy. Thus, precise and timely molecular subtype classification is critical for optimizing individualized treatment strategies.

While Imaging techniques such as magnetic resonance imaging (MRI), ultrasound, and mammography are widely used in breast cancer detection (5), core needle biopsy (CNB) with immunohistochemistry (IHC) remains the diagnostic gold standard for molecular classification. However, CNB is invasive, costly, time-intensive, and may fail to capture tumor heterogeneity, potentially leading to diagnostic inaccuracies (6). Radiomics, an emerging technology providing quantitative tumor assessments beyond human visual interpretation, offers promising solutions to these challenges (7, 8).

MRI-based radiomics has effectively evaluated malignancy and molecular subtypes (9–11). Free from ionizing radiation, ultrasound is particularly suitable for young and pregnant women and demonstrates higher sensitivity than mammography for detecting intraductal and nodular lesions (12). In populations with smaller, denser breasts, such as younger and Chinese women, ultrasound is preferred for breast lesion screening and preoperative evaluation (13). However, grayscale ultrasound’s dependence on radiologist interpretation introduces variability and subjectivity, prompting the development of radiomics-based approaches to address these limitations but are frequently confined to the single modality of two-dimensional (2D) ultrasound (14–17). Recent advances in deep learning (e.g., ResNet-101) show promise in unimodal ultrasound classification (18), yet remain constrained by single-modality data. Multimodal fusion may address critical diagnostic gaps in clinical practice, particularly for lesions with ambiguous imaging phenotypes (19).

Advancements in ultrasound technology have introduced modalities such as CDFI, SE, and ABVS, which offer distinct advantages in breast lesion assessment (20, 21). CDFI can quantify the vascular distribution of tumors, and SE is capable of measuring tissue hardness. Existing studies have shown that both elastography and CDFI are correlated with the Luminal A cancer subtype (22). ABVS, in particular, enables coronal imaging that enhances the visualization of lesions and adjacent tissues (23), facilitating detailed characterization and improved assessment of tissue architecture and spatial relationships. Additionally, ABVS produces standardized images, minimizing variability and making it ideal for radiomics analysis, where consistency and reproducibility are key for extracting meaningful features (24).

ABVS coronal combined with SE has demonstrated efficacy in distinguishing breast lesions (25), but radiomics studies utilizing ABVS for molecular subtype classification are still scarce. Manual feature selection methods, commonly used in healthcare data analysis, are inadequate for handling the growing complexity of multimodal imaging data. Unstable selection processes can lead to, substantial variability in selected feature subsets, compromising model reliability (26, 27).

This study aims to integrate multiple ultrasound modalities—including 2D ultrasound, CDFI, SE, and ABVS coronal imaging—into a machine-learning framework designed to preoperatively differentiate luminal from non-luminal breast cancer subtypes. Unlike prior research predominantly focused on single-modality radiomics, this study adopts a multimodal fusion approach to achieve superior accuracy and reliability in subtype differentiation. To further enhance model stability and performance, diverse feature selection strategies are employed to address the limitations of unstable selection methods, thereby improving the overall robustness and reliability of the analysis.

## 2 Methodology

This retrospective study included 247 breast cancer cases diagnosed through histopathological evaluation of CNB samples or surgical specimens at our hospital between January 2020 and June 2024. Ethical approval was obtained from the hospital review board, and informed consent was waived. Patient information was anonymized to ensure privacy.

**Figure 1:**
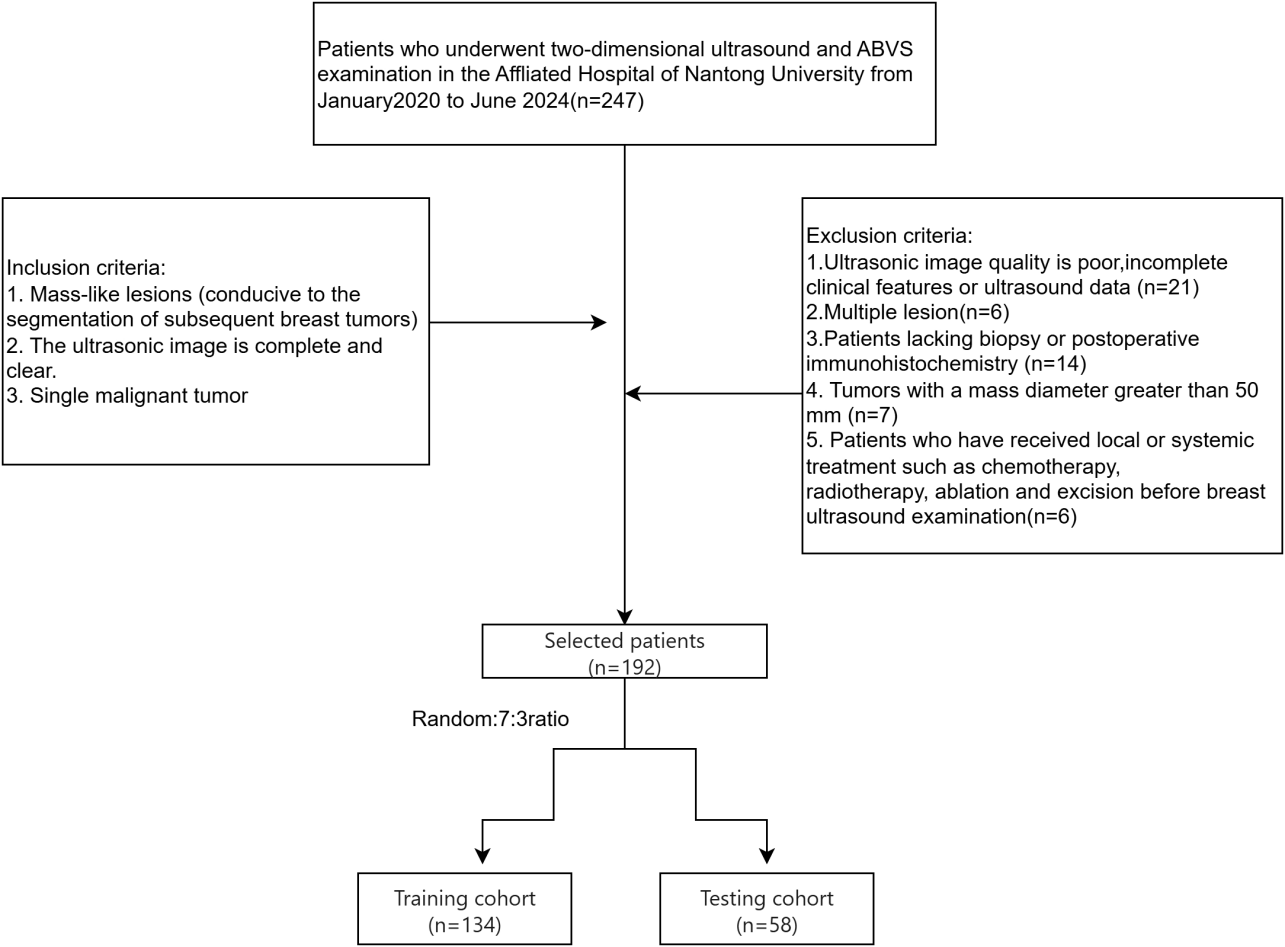
Flowchart illustrating the selection process for breast cancer patients, detailing the inclusion and exclusion criteria and the allocation to training and testing cohorts.

The inclusion criteria were (1): complete and high-quality ultrasound images with mass-like lesions suitable for tumor segmentation and (2) solitary malignant tumors. Exclusion criteria included (1): poor-quality ultrasound images, (2) multiple lesions, (3) incomplete clinical or ultrasound data, (4) lack of puncture or surgical IHC results, (5) tumor diameters exceeding 50 mm(Large tumors frequently exceed standard ultrasound probe fields-of-view, resulting in discontinuous ROI segmentation that compromises radiomic feature stability.), and (6) prior local or systemic therapy (e.g., chemotherapy, radiation, ablation, or excision) before breast ultrasonography. Of the 247 cases, 192 patients met the criteria, including 140 ductal and 52 non-ductal breast cancers. Patients were randomly divided into a test set (134 cases) and a validation set (58 cases) in a 7:3 ratio (**Figure 1**).

### 2.1 Clinical information

Clinical data collected for training and validation sets included age, ultrasound-reported tumor size, microcalcifications, convergence sign, breast imaging reporting and data system (BI-RADS) classification, pathology, strain elasticity score, ER/PR/HER2 status, Ki-67, and molecular subtype. Tumors were classified as luminal or non-luminal based on hormone receptor (HR) status from IHC results. Tumors with ≥1% of cells staining positive for ER or PR were categorized as luminal, while HR-negative tumors were classified as non-luminal (28). Images were labeled accordingly.

### 2.2 Image acquisition

Breast ultrasound examinations were performed by two experienced physicians, each with over 5 years of expertise in breast imaging. A Siemens Acuson Oxana 2 ABVS (Siemens Healthineers, Erlangen, Germany) equipped with 9L4 and 14L5B line-array probes performing radial, transverse, and longitudinal scans. The largest ultrasonic area was evaluated, and ultrasound features such as BI-RADS classification, size, location, shape, margins, internal echoes, microcalcifications, strain elasticity score, convergence sign, vascularity, and axillary lymph node involvement were documented. All modalities were acquired consecutively within 2 hours using the same scanner (Siemens Acuson Oxana 2). ROIs were segmented from synchronized ABVS/2D-US images.

### 2.3 Image segmentation and feature extraction

**Figure 2:**
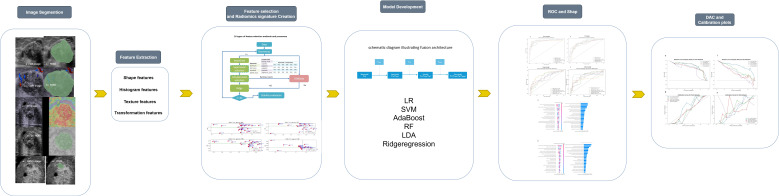
Workflow diagram of the study, outlining the key steps from data acquisition to model evaluation.

**Figure 2** outlines the radiomics workflow, including segmentation, feature extraction and selection, image preprocessing, feature analysis, and model construction. Image preprocessing was performed following IBSI(Imaging Biomarker Standardization Initiative) guidelines, including voxel resampling for spatial normalization, intensity discretization, and z-score normalization of feature values. The target area was delineated using the open-source software 3D Slicer (version 5.6.1), and features—such as first-order, morphological, grayscale histogram, and wavelet transforms—were extracted using the SlicerRadiomics extension.

Forty ultrasound images (20 luminal and 20 non-luminal cases) were randomly selected (29), with regions of interest (ROIs) independently delineated by two physicians for intergroup consistency. Two weeks later, Physician 1 repeated the ROI delineation for intragroup consistency testing. The intraclass correlation coefficients (ICCs) for intergroup and intragroup tests exceeded 0.75, confirming high feature consistency. Subsequently, Physician 1 segmented the remaining images, retaining only features with an ICC above 0.75 for further analysis.

### 2.4 Feature selection and dataset construction

**Figure 3:**
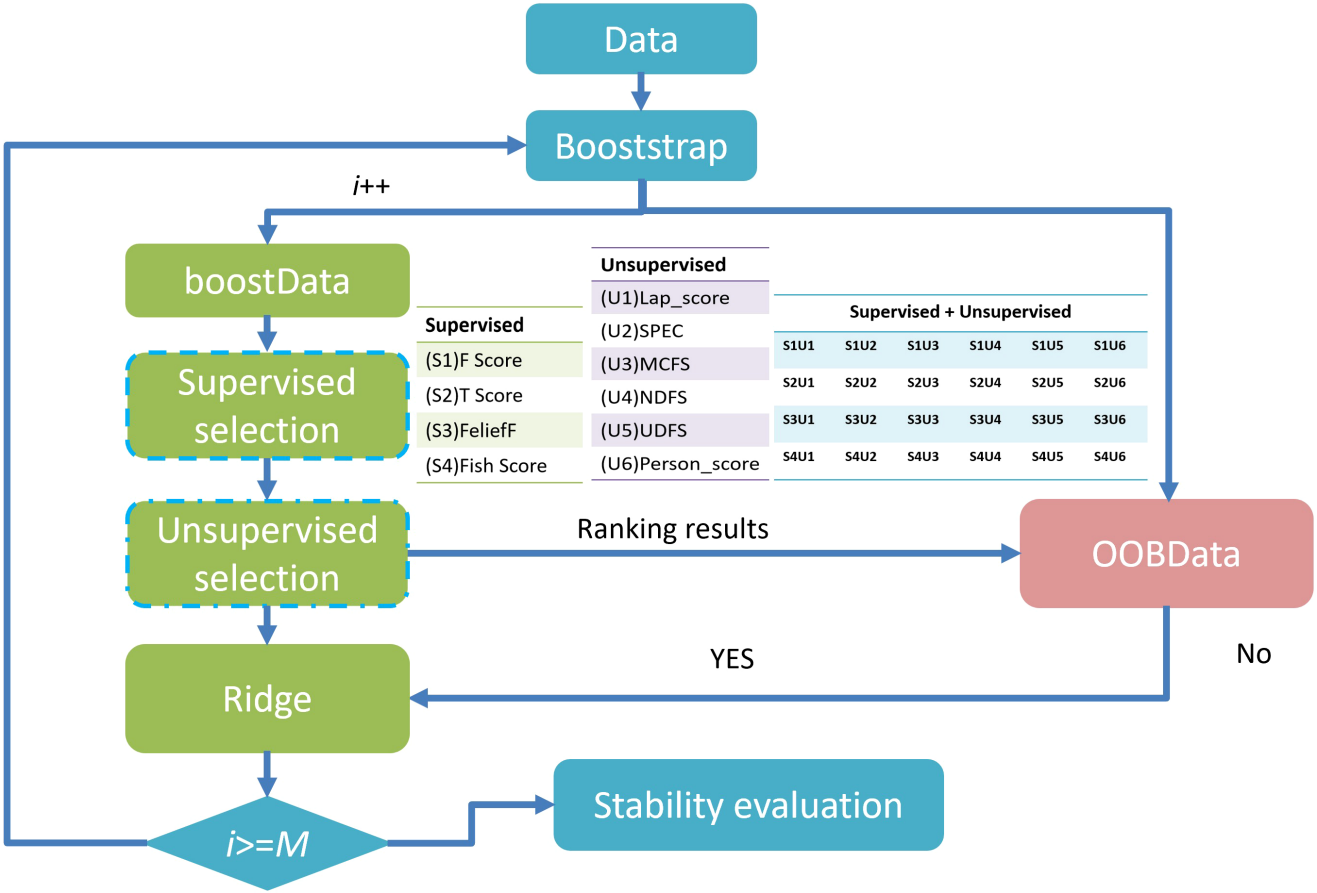
Overview of feature selection methods and workflow.

When the number of patients is substantially smaller than the number of extracted features, the data becomes sparse within a high-dimensional space (26), undermining machine learning models’ accuracy and generalizability. To reduce redundancy, exclude irrelevant features, and minimize the risk of overfitting, the method proposed by Li et al. (30) was employed, incorporating six unsupervised feature selection (FS) algorithms—Lap_score, SPEC, MCFS, NDFS, UDFS, and person score—alongside four supervised FS algorithms—F score, Tscore, ReliefF, and Fish_score— yielding a total of 24 FS combinations. Supervised FS: Depends on data labels (such as the categories in classification tasks and the continuous values in regression tasks). The goal is to select features that make the model more effective in predicting labels. Unsupervised FS: Does not rely on labels. The goal is to optimize the internal structure of the feature set (such as reducing redundancy and retaining key patterns). It is often used for subsequent unsupervised tasks (like clustering, dimensionality reduction) or as a preprocessing step for supervised tasks. After evaluation, we finally selected the dataset with AUC(Area Under the Curve)*stability greater than 0.45 for subsequent analysis. Feature selection was independently conducted for each dataset grouping to eliminate potential cross-group interference and to preserve methodological integrity. The overarching FS strategy is illustrated in **Figure 3**. This process facilitated the generation of multiple datasets with increasing complexity. These included monomodal datasets comprising 2D US images, dual-modal datasets integrating 2D US with CDFI (US+CDFI), trimodal datasets incorporating SE alongside 2D US and CDFI (US+CDFI+SE), and four-modal datasets combining all modalities, including ABVS coronal imaging (US+CDFI+SE+ABVS).

Feature fusion was performed by channel concatenation:


Ffused​=[FUS​∣FCDFI​∣FSE​∣FABVS​]

where $FUS\in R^{n}\times 15,FCDFI\in R^{n}\times 15,FSE\in R^{n}\times 15,FABVS\in R^{n}\times 15$ … denote modality-specific feature matrices(e.g., ultrasound, color Doppler, shear wave elastography, etc.), ∣ represents column-wise concatenation (stacking matrices along the feature channel dimension), and *n* is the sample size (number of data instances).

### 2.5 ML model derivation and validation

Six classifiers—LR, AdaBoost, LDA, ridge regression, SVM, and RF—were used for prediction. Synthetic Minority Over-sampling Technique (SMOTE) oversampling balanced the dataset, and 10-fold cross-validation with grid search was used for hyperparameter optimization(e.g. SVM (kernel: linear/RBF; penalty C: [0.1, 1, 10]; γ: [0.001, 0.01, 0.1]; optimal: RBF kernel, C = 1, γ=0.01)). This approach reduces evaluation bias caused by uneven training data division, providing a more stable performance assessment. The Bootstrap method was used to calculate the confidence interval of AUC, and the Dunn-Sidak correction was applied to control the multiple comparison errors. All workflows were implemented in Python (version 3.8). Calibration curves assessed alignment between predictions and outcomes, while decision curve analysis (DCA) evaluated clinical utility. Calibration curves were generated by binning predicted probabilities (10 quantile bins) and plotting mean predictions against observed event rates, with perfect calibration indicated by a 45° line.

### 2.6 SHAP model

Using game theory principles, SHAP (Shapley Additive Explanations) quantified each feature’s contribution to the model’s output. This interpretability framework visualized feature importance, highlighting the impact of individual variables on predictions (31). We applied SHAP values to assess each feature’s contribution to the optimal model and its influence on decision-making in specific scenarios.

### 2.7 Model evaluation and statistical analysis

Continuous variables were analyzed using the Mann-Whitney U or Student’s t-test, depending on normality assumptions, while Pearson’s chi-square test assessed categorical differences. Model performance metrics included AUC, sensitivity, specificity, positive and negative predictive values, and F1-score. Statistical significance was set at p< 0.05.

## 3 Results

### 3.1 Patient cohort distribution

**Table 1** summarizes the characteristics of patients in the training and validation cohorts, revealing no statistically significant differences in age, tumor size, microcalcification, ultrasound convergence signs, BI-RADS classification, pathological tumor type, SE score, or molecular subtype between the two groups (p > 0.05).

**Table 1:** Comparative analysis of demographic and clinical parameters across the training and testing cohorts.

Characteristics		Training set (count, %)	Validation set (count, %)	P-value
Age (y)		56.50 [50.00, 63.00]	56.50 [49.25, 67.75]	0.398
Tumor size on US (mm)		2.05 [1.50, 2.50]	2.05 [1.50, 3.08]	0.393
BI-RADS	4A	13 (9.7%)	4 (6.9%)	0.865
	4B	31 (23.1%)	12 (20.7%)	
	4C	64 (47.8%)	31 (53.4%)	
	5	26 (19.4%)	11 (19.0%)	
Convergence sign	Yes	69 (51.5%)	24 (41.4%)	0.258
	No	65 (48.5%)	34 (58.6%)	
Microcalcifications	Yes	63 (47.0%)	24 (41.4%)	0.574
	No	71 (53.0%)	34 (58.6%)	
Histologic type	IDC	93 (69.4%)	39 (67.2%)	0.138
	ILC	24 (17.9%)	6 (10.3%)	
	Other*	17 (12.7%)	13 (22.4%)	
ER status	Positive	99 (73.9%)	39 (67.2%)	0.444
	Negative	35 (26.1%)	19 (32.8%)	
PR status	Positive	77 (57.5%)	31 (53.4%)	0.722
	Negative	57 (42.5%)	27 (46.6%)	
HER2 status	Positive	111 (82.8%)	49 (84.5%)	0.944
	Negative	23 (17.2%)	9 (15.5%)	
Molecular subtypes	Luminal	101 (75.4%)	39 (67.2%)	0.323
Molecular subtypes	Non-luminal	33 (24.6%)	19 (32.8%)	
Elasticity	2	1 (0.7%)	0 (0.0%)	0.643
	3	17 (12.7%)	11 (19.0%)	
	4	32 (23.9%)	13 (22.4%)	
	5	84 (62.7%)	34 (58.6%)	
Ki-67	_=_14	120 (89.6%)	45 (77.6%)	0.051
	<14	14 (10.4%)	13 (22.4%)	

Age and tumor size on US were presented as median [range]. All other data are presented as counts (percentages). The Kruskal-Wallis rank sum test was used to compare age, US tumor size, and BI-RADS classification. The chi-square test was used to compare ER, PR, HER2, Ki-67 status, and molecular subtypes.

* Other, just one histologic type other than the IDC or ILC

* Non-luminal subtypes: HER2-enriched (n=14), TNBC (n=5) in test set

*US, ultrasound; BI-RADS, breast imaging reporting and data system; IDC, Invasive ductal cancer; ILC, Invasive lobular cancer; ER, estrogen receptor; PR, progesterone receptor; HER2, human epidermal growth factor receptor 2*

### 3.2 Radiomics feature extraction and selection

**Figure 4:**
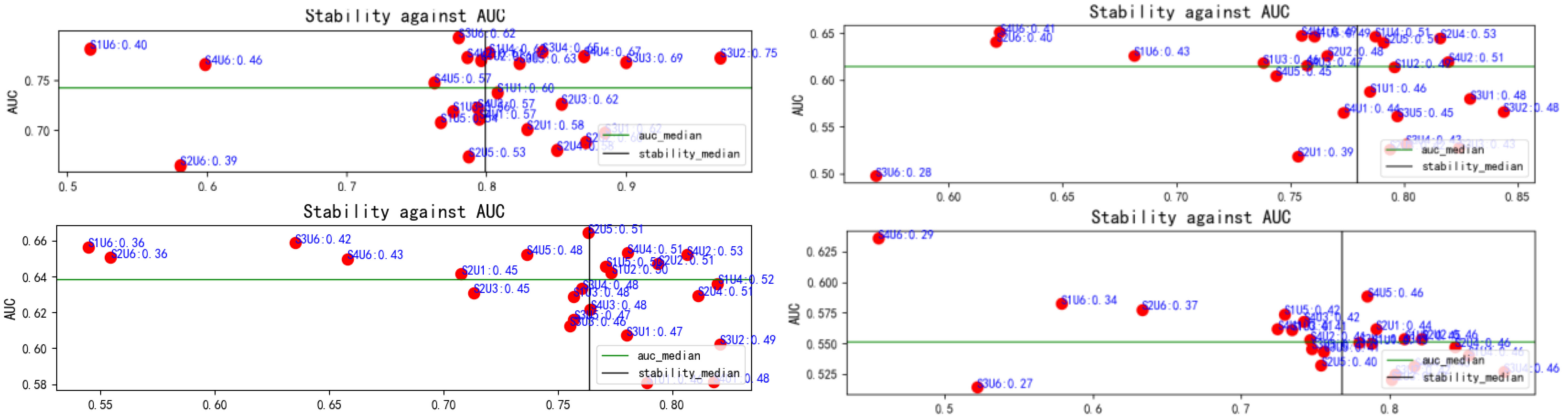
Relationship between feature selection method stability and AUC. Red dots represent the results of each method, with stability plotted on the x-axis and AUC on the y-axis. The green and black lines indicate the median AUC and stability, respectively. This figure highlights variations in stability and classification performance across methods. AUC, area under the curve.

For each patient, 863 features were uniformly extracted from the ROIs across the four imaging modalities: US, CDFI, SE, and ABVS coronal imaging. These features included radiomics metrics such as gray-level co-occurrence matrices (GLCM) and gray-level dependence matrices (GLDM). During feature selection, 24 distinct methods were applied to independently screen features within each modality, resulting in a 192 x 15 unimodal dataset for each modality (**Figure 4**). Optimal feature selection combinations included Fish score and UDFS, T-score and UDFS, F score and NDFS, and ReliefF and NDFS. The data from each modality were then fused at the feature level to generate multimodal datasets corresponding to dimensions of 192 x 15 (monomodal), 192 x 30 (dual-model), 192 x 45 (trimodal), and 192 x 60 (four-modal).

### 3.3 ML model derivation and evaluation

**Figure 5:**
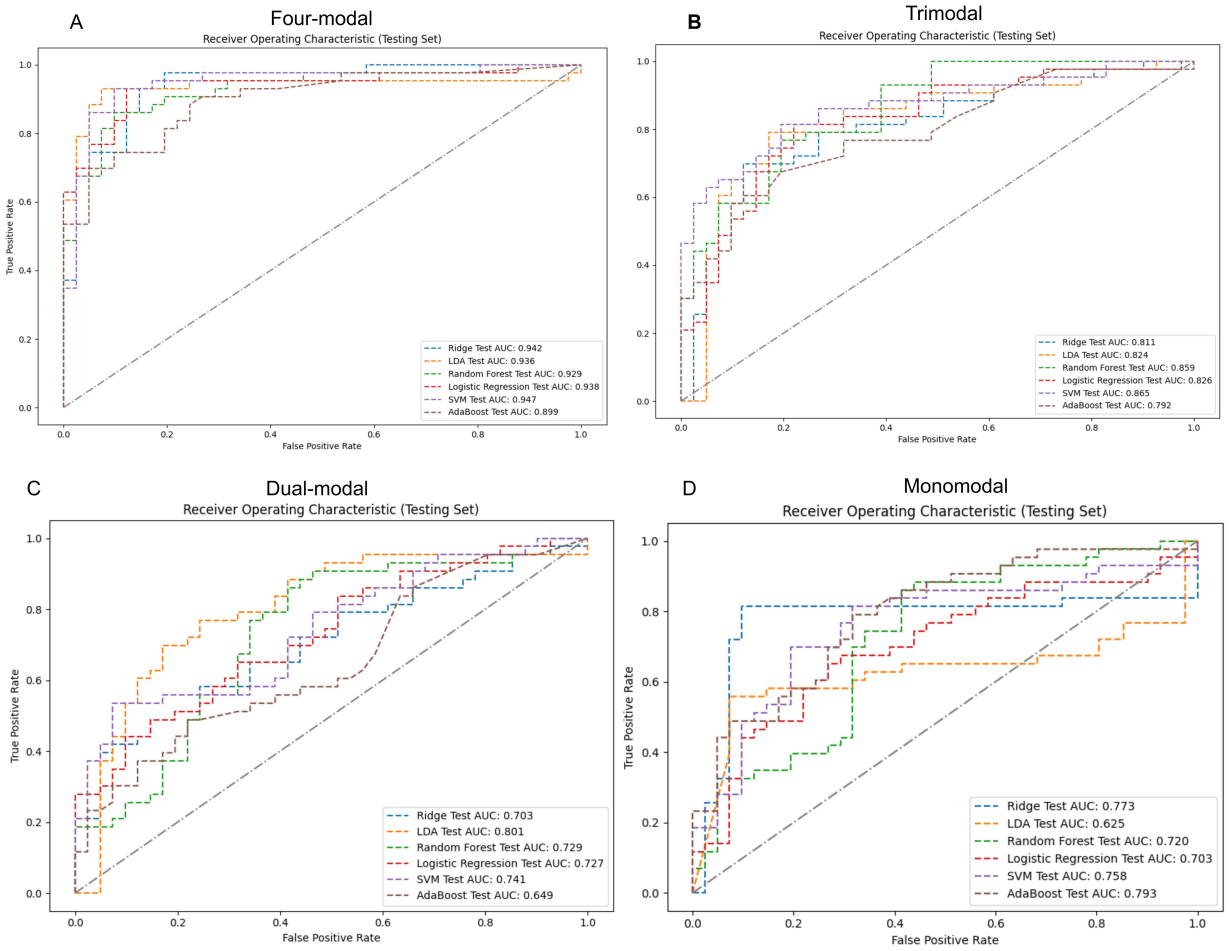
ROC curves and AUC values for various modal models using machine learning classifiers on the test set. Panels **(A-D)** sequentially present the performance of monomodal, dual-modal, trimodal, and four-modal models. AUC, area under the curve; ROC, receiver operating characteristic; SVM, support vector machine; AdaBoost, adaptive enhancement; LDA, linear discriminant analysis; Ridge, ridge regression.

Four-modal models demonstrated significantly better performance in the test set than monomodal models across all machine learning algorithms (**Figure 5**). The SVM four-modal model achieved the highest performance, with an AUC of 0.947 (95% CI: [0.884, 0.986]), which was higher than the AUC of 0.758 (95% CI: [0.637, 0.853]) observed in the monomodal model. Other algorithms, including AdaBoost, LR, RF, and ridge regression, also achieved higher AUC values in four-modal configurations than monomodal ones. The trimodal SVM model had an AUC of 0.865 (95% CI: [0.778, 0.938]), which was higher than that in the dual-modal (AUC 0.741, 95% CI: [0.648, 0.854]) and monomodal models (AUC 0.758, 95% CI: [0.637, 0.853]). The specificity and sensitivity of the trimodal model were higher than those of the dual-modal and monomodal models. However, both metrics remained lower than those observed in the four-modal model, indicating improved diagnostic performance.

**Figure 6:**
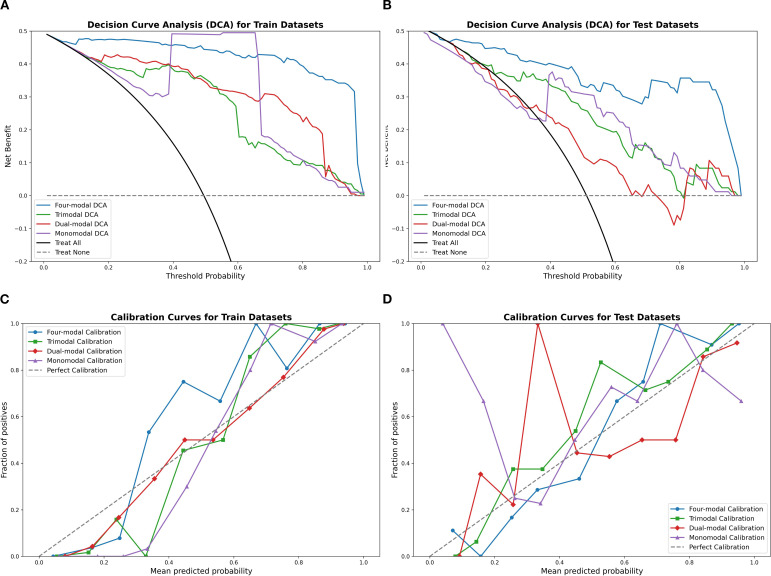
Calibration curves and DCA results using the SVM classifier, comparing the four-modal, trimodal, dual-modal, and monomodal models. Panels **(A, B)** display calibration curves for the training and test sets, respectively, while panels **(C, D)** present the DCA results. The four-modal model demonstrates superior calibration accuracy and clinical decision-making utility. SVM, support vector machine; DCA, decision curve analysis.

**Table 2** highlights the superior predictive performance of four-modal models across all machine learning algorithms. The AUC of the SVM four-modal model (0.947 (95% CI: [0.884, 0.986]) was higher than that in any other configuration. The LR four-modal model achieved the highest sensitivity (0.884, 95% CI: [0.778, 0.976]), specificity (0.927, 95% CI: [0.841, 1.000]), and F1-score (0.905, 95% CI: [0.829, 0.965]). Calibration curves and DCA confirmed the accuracy and clinical applicability of the four-modal models (**Figure 6**). Overall, these findings demonstrate that multimodal fusion significantly enhances the predictive capacity of each model.

**Table 2:** Predictive performance of SVM and LR models in classifying luminal breast cancer subtypes.

Train	AUC	Sensitivity (%)	Specificity (%)	PPV (%)	NPV (%)	F1 (%)
Four-modal						
SVM	0.990(0.978,0.998)	0.897(0.835,0.955)	0.970(0.927,1.000)	0.967(0.922,1.000)	0.906(0.845,0.957)	0.930(0.886,0.964)
LR	0.997(0.992,1.000)	0.969(0.931,1.000)	0.980(0.947,1.000)	0.979(0.948,1.000)	0.970(0.931,1.000)	0.974(0.947,0.995)
Trimodal						
SVM	0.981(0.965,0.993)	0.887(0.820,0.943)	0.949(0.904,0.989)	0.945(0.894,0.988)	0.895(0.832,0.948)	0.915(0.872,0.952)
LR	0.944(0.911,0.971)	0.866(0.794,0.932)	0.879(0.810,0.939)	0.875(0.806,0.937)	0.870(0.802,0.935)	0.870(0.816,0.918)
Dual-modal						
SVM	0.936(0.900,0.967)	0.804(0.716,0.882)	0.889(0.827,0.951)	0.876(0.806,0.942)	0.822(0.743,0.893)	0.839(0.777,0.891)
LR	0.834(0.777,0.890)	0.711(0.626,0.796)	0.808(0.730,0.880)	0.784(0.699,0.867)	0.741(0.661,0.824)	0.746(0.678,0.810)
Mono-modal						
SVM	0.973(0.945,0.996)	0.938(0.888,0.981)	0.939(0.892,0.981)	0.938(0.888,0.980)	0.939(0.891,0.981)	0.938(0.901,0.971)
LR	0.998(0.996,1.000)	0.979(0.946,1.000)	0.980(0.949,1.000)	0.979(0.950,1.000)	0.980(0.948,1.000)	0.979(0.956,0.995)
Test	AUC	Sensitivity (%)	Specificity (%)	PPV (%)	NPV (%)	F1 (%)
Four-modal						
SVM	0.947(0.884,0.986)	0.791(0.667,0.902)	0.927(0.833,1.000)	0.919(0.824,1.000)	0.809(0.681,0.917)	0.850(0.754,0.925)
LR	0.938(0.863,0.983)	0.884(0.778,0.976)	0.927(0.841,1.000)	0.927(0.846,1.000)	0.884(0.775,0.976)	0.905(0.829,0.965)
Trimodal						
SVM	0.865(0.778,0.938)	0.674(0.531,0.818)	0.878(0.766,0.973)	0.853(0.719,0.968)	0.720(0.592,0.842)	0.753(0.630,0.854)
LR	0.826(0.763,0.925)	0.721(0.581,0.850)	0.829(0.697,0.938)	0.816(0.690,0.931)	0.739(0.605,0.857)	0.765(0.650,0.854)
Dual-modal						
SVM	0.741(0.648,0.854)	0.558(0.405,0.696)	0.854(0.737,0.953)	0.800(0.636,0.933)	0.648(0.509,0.772)	0.658(0.521,0.775)
LR	0.727(0.681,0.884)	0.674(0.525,0.805)	0.780(0.639,0.900)	0.763(0.621,0.897)	0.696(0.553,0.824)	0.716(0.593,0.818)
Mono-modal						
SVM	0.758(0.637,0.853)	0.605(0.452,0.750)	0.805(0.684,0.919)	0.765(0.625,0.897)	0.660(0.521,0.800)	0.675(0.540,0.786)
LR	0.703(0.618,0.846)	0.558(0.415,0.703)	0.805(0.683,0.917)	0.750(0.600,0.897)	0.635(0.500,0.767)	0.640(0.507,0.761)

Data in parentheses are 95% confidence intervals.

*LR, logistic regression; SVM, support vector machine; AUC, area under the curve; PPV, positive predictive value; NPV, negative predictive value; F1, F1-score.*

### 3.4 Model interpretation (SHAP)

**Figure 7:**
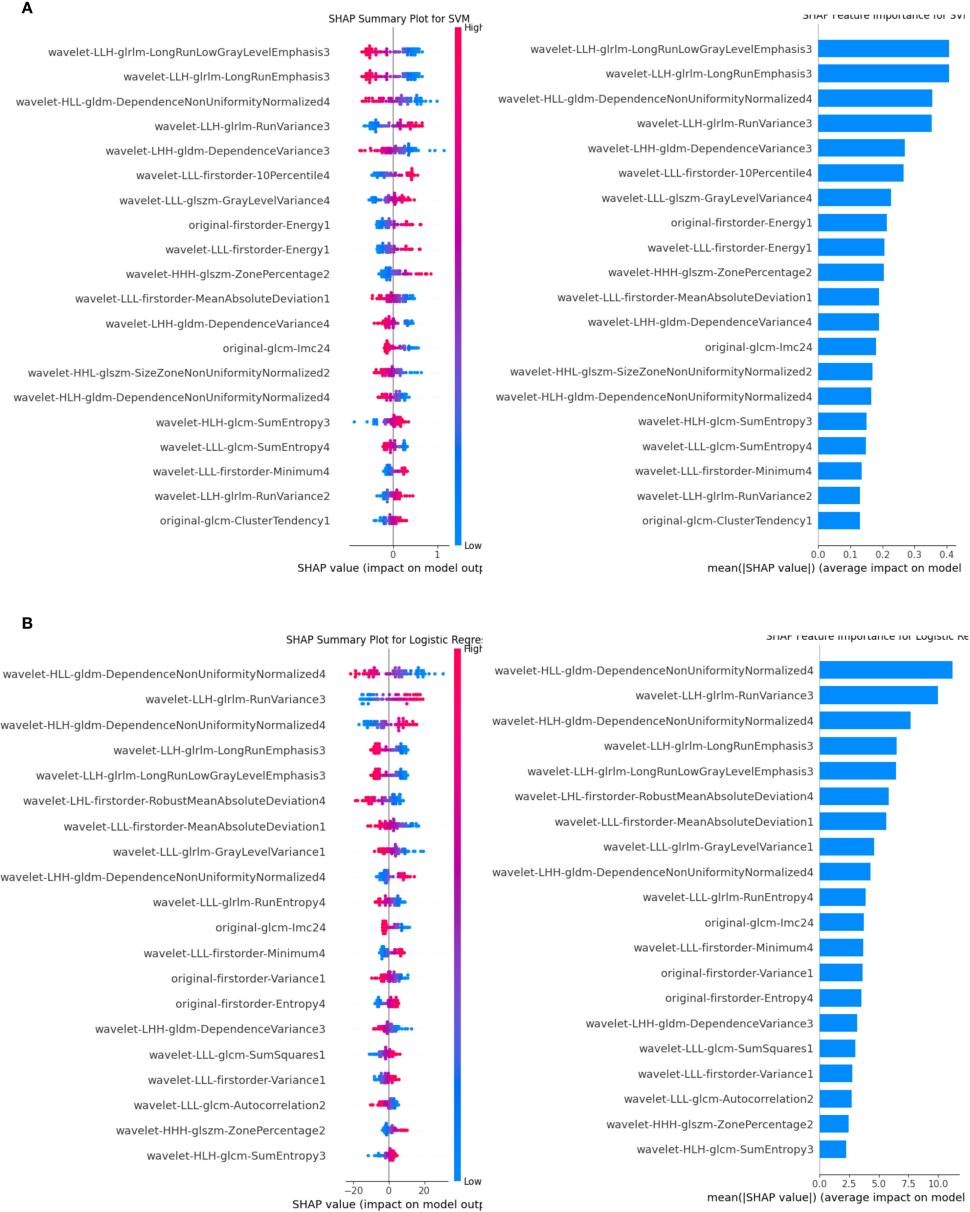
SHAP analysis for the SVM and LR classifiers. **(A)** illustrates the SVM results, while **(B)** shows the LR results. Features are ranked by their mean absolute SHAP values, with top-ranked features significantly impacting predictions. The summary plot visualizes how feature values influence model output, with blue representing lower feature values and red representing higher feature values. The horizontal position of each dot reflects the SHAP value, indicating the feature’s effect on individual predictions. LR, logistic regression; SVM, support vector machine; SHAP, Shapley Additive Explanations.

The SVM and LR four-modal models demonstrated superior performance based on a comprehensive evaluation. SHAP analysis quantified the contribution of each feature, with average absolute SHAP values serving as the primary metric. **Figure 7** visualizes the cumulative impact of each feature, where higher eigenvalues (in red) indicate a stronger positive influence on predictions. In Figure I, the features wavelet-LLH-glrlm-LongRunLowGrayLevelEmphasis3 and wavelet-LLH-glrlm-LongRunEmphasis3 had a greater influence on predictions in the SVM model than other features. In contrast, Figure G shows that wavelet-HLL-gldm-DependenceNonUniformityNormalized4 was the most significant feature for predictions in the LR model, followed by wavelet-LLH-glrlm-RunVariance3. Overall, features belonging to GLDM (Gray Level Dependency Matrix), GLRLM (Gray Level Run Length Matrix), GLSZM (Gray Level Size Zone Matrix), first-order statistical features, and wavelet features contributed the most to the predictions in both models.

## 4 Discussion

This study successfully developed a multimodal machine learning model to classify luminal and non-luminal breast cancer subtypes preoperatively. The progressive integration of modalities—from 2D-US to US+CDFI+SE+ABVS—yielded significant AUC gains (monomodal:0.758 → four-modal:0.947, Δ+0.189). This demonstrates that combining structural and functional data outperforms single-modality assessment. These findings underscore the critical role of multimodal data fusion in enhancing model generalizability and accuracy. Given the diagnostic challenges posed by tumor heterogeneity and biopsy limitations, this approach provides a promising complementary tool for personalized treatment strategies in early-stage breast cancer.

As non-luminal breast cancer often necessitates neoadjuvant therapy, the developed model provides a promising complementary tool for personalized treatment strategies in patients with early-stage breast cancer. Tumor heterogeneity, which can lead to inaccurate IHC results from preoperative biopsies, highlights the value of imaging-based approaches for molecular subtype classification (32, 33). Radiomics techniques have effectively addressed diagnostic gaps inherent in biopsy limitations, supporting their integration into clinical workflows.

Previous studies have confirmed the potential of mammographic radiomics and MRI-based analyses in predicting molecular subtypes. Early studies reported AUC values up to 0.836 (34, 35), while recent research advancements have shown that mammographic radiomics models (36) have achieved AUC values of 0.855, and multi-parametric MRI (mpMRI)-based feature fusion models (37) have reported AUCs of over 0.81.These findings indicate that advanced imaging techniques hold significant potential as effective complementary tools for accurately classifying breast cancer molecular subtypes, thereby augmenting existing diagnostic approaches. In comparison, ABVS offers distinct advantages by combining standard ultrasound and ABVS imaging into a single examination, thereby improving clinical efficiency, diagnostic accuracy, and patient management (38–40).

Despite its utility, the correlation between ABVS coronal imaging and breast cancer molecular subtypes remains underexplored. This study addresses this gap by integrating ABVS with other imaging modalities, demonstrating its effectiveness in multimodal classification.

Our study demonstrated that LR and SVM models were the most effective in distinguishing between luminal and non-luminal breast cancers, consistent with findings from prior research. For instance, a study utilizing 2D ultrasound to classify PR-positive and PR-negative breast cancers achieved an AUC of 0.879 for the LR classifier, surpassing the performance of our monomodal model (41). This disparity may be attributed to the larger dataset size in the prior study, which likely enhanced the generalizability of the model in distinguishing between different molecular subtypes. Our multimodal approach demonstrated improved performance in our cohort, particularly with the SVM model. The SVM’s ability to handle complex nonlinear relationships through its kernel method enabled more accurate classification, underscoring its suitability for multimodal datasets (42, 43).

As Jiangfeng Wu et al. noted, texture, wavelet, and first-order features are crucial for differentiating luminal and non-luminal subtypes, with features such as wavelet-HLL-gldm-DependenceNonUniformityNormalized, original-glrlm-HighGrayLevelRunEmphasis, and wavelet-LHL-glszm-SizeZoneNonUniformity showing particular significance. Shape-based features, however, were omitted due to their lower diagnostic relevance (17). Our findings align closely with these observations and suggest that refining the extraction and application of these features could further enhance diagnostic accuracy.

The multimodal model showed strong performance for HER2-enriched subtypes (n=14 in test set), but TNBC predictions (n=5) require validation due to limited samples. Caution is warranted when generalizing TNBC results. The SVM and LR classifiers demonstrated outstanding performance within our multimodal model, particularly when ABVS and SE images were incorporated. During the feature selection process, the majority of the ten most important features originated from ABVS and SE images, while fewer key features were derived from CDFI images. These results highlight the pivotal role of ABVS and SE imaging in accurately classifying molecular subtypes of breast cancer, emphasizing their integration as essential components of multimodal radiomics workflows.

The Random Forest (RF) model's perfect AUC of 1.0 on the unimodal training set strongly indicates overfitting. Furthermore, the integration of a second modality did not consistently yield improvements; conversely, the dual-modal configuration (US+CDFI) exhibited a marginally lower AUC than the monomodal US in some classifiers (e.g., SVM: 0.741 vs. 0.758). We attribute this to feature redundancy between vascularity (CDFI) and parenchymal texture (US), which introduced noise without augmenting discriminatory power. Nevertheless, incorporating SE and ABVS coronal imaging resolved this limitation by adding orthogonal biological information—tissue stiffness and 3D architectural distortion—yielding significant improvements in four-modal models. Overfitting typically occurs when models are trained on a limited set of features, resulting in the fitting of noise within the training data and compromised performance on the unseen test data. In this study, the multimodal model leveraged a more diverse and comprehensive feature set by integrating multiple imaging modalities, thereby improving robustness and stabilizing metrics such as AUC. These findings underscore the value of multimodal fusion in mitigating overfitting and enhancing the generalizability of machine learning models for complex diagnostic tasks.

The clinical value of this model may extend beyond diagnostic accuracy to optimizing diagnostic and therapeutic pathways. For instance, in patients with a high-confidence prediction of a low-risk Luminal A-type profile (e.g., predicted probability >0.90), the model could potentially support a discussion about proceeding directly to surgery, with definitive diagnosis and full molecular subtyping (including Ki-67 and HER2 status) confirmed postoperatively on the surgical specimen. However, it is crucial to emphasize that this approach would be entirely inappropriate for cases where clinical or imaging features suggest a more aggressive phenotype, or specifically for patients who are potential candidates for neoadjuvant therapy (e.g., those with Luminal B2/HER2+ or triple-negative subtypes). In these scenarios, a core needle biopsy remains the absolute standard of care to obtain essential biomarker information (most critically, HER2 status) necessary to guide neoadjuvant treatment decisions. For patients contraindicated for biopsy (e.g., coagulation disorders), the model could provide a non-invasive risk assessment to aid in clinical planning. Overall, the model is intended as a complementary decision-support tool, not a replacement for standard pathological diagnosis.

Future investigations should prioritize the refinement of feature extraction and selection methodologies to further enhance the diagnostic precision of multimodal models. Evaluating the impact of different modality combinations on model performance and comparing their respective AUC values could inform strategies to optimize clinical workflows, reduce examination burdens, and improve diagnostic efficiency. These advancements would bolster the efficacy of multimodal fusion and provide clinicians with streamlined and reliable tools for decision-making in complex oncologic cases.

This study has inherent limitations. Conducting the research within a single facility and relying on a relatively small sample size restricts the generalizability of these findings, underscoring the necessity of validating the model in larger, multicenter cohorts. To address the lack of external validation, we implemented rigorous cross-validation procedures. However, obtaining four-modal external validation datasets proved particularly challenging. Despite contacting multiple hospitals, insufficient sample sizes were available. We plan to expand our collaborative efforts to collect multi-institutional validation cohorts in future studies. Additionally, manual delineation of tumor ROIs by sonographers introduced potential variability due to subjective interpretation. While inter-observer agreement assessments partially mitigate this concern, future studies should explore automated segmentation techniques to reduce operatory bias and ensure reproducibility. Finally, the dataset imbalance, with non-luminal breast cancers (e.g., HER2-enriched and triple-negative types) comprising a smaller proportion of cases, necessitated oversampling, which may have introduced additional complexities. A comprehensive evaluation of all available machine learning algorithms was beyond the scope of this study and represents a worthwhile area for future research.

The deployment of this study across hospitals using diverse ultrasound platforms faces three primary barriers: inter-system variability—where differences in image resolution, dynamic range, and post-processing algorithms (e.g., Siemens vs. GE vs. Philips systems) may alter radiomic feature values and compromise model generalizability; lack of protocol standardization—where variations in scanning parameters (e.g., frequency, depth, gain settings) and operator-dependent acquisition techniques (e.g., probe pressure variations during SE) introduce feature instability; and ABVS dependence—given the model’s heavy reliance on ABVS coronal features (Section 3.4, SHAP analysis), leading to reduced accuracy in trimodal/four-modal configurations at hospitals without ABVS capabilities. To address these challenges, future work will focus on developing vendor-agnostic feature normalization pipelines, establishing IBSI-compliant imaging acquisition guidelines, and exploring domain adaptation techniques to align heterogeneous data distributions.

### 4.1 Conclusion

This study developed a multimodal machine learning model for preoperative differentiation of luminal and non-luminal breast cancer subtypes. By incorporating ABVS and SE, the model demonstrated enhanced generalization and predictive accuracy, highlighting the clinical value of multimodal ultrasound imaging. However, external validation in diverse populations is essential prior to clinical deployment.

## Data Availability

The data analyzed in this study is subject to the following licenses/restrictions: No, the datasets are not included in the article/supplementary material to protect participant privacy. Requests to access these datasets should be directed to Yan Fu, 1159449021@qq.com.
